# Preliminary analysis of whether mosquitoes can carry and transmit African swine fever

**DOI:** 10.1186/s12917-021-02865-2

**Published:** 2021-04-09

**Authors:** Weiyun Qin, Zhongcheng Gao, Shenglong Wu, Wenbin Bao

**Affiliations:** 1 College of Animal Science and Technology, Yangzhou University, 225009 Yangzhou, China; 2 Joint International Research Laboratory of Agriculture & Agri-Product Safety, Yangzhou University, 225009 Yangzhou, China

**Keywords:** African swine fever, Mosquito, Probe‐based qPCR, Insect vector, Virus transmission, Pig farms

## Abstract

**Background:**

Mosquitoes are important insect vectors, but whether they can carry and transmit African swine fever virus (ASFV) in large-scale pig farms in China is unknown.

**Results:**

In this study, probe-based qPCR analysis was performed on mosquitoes from five pig farms with ASF virus (ASFV). Analysis of ASFV in 463 mosquitoes yielded negative cycle threshold (CT) value), and detection remained negative after mixing samples from all five pig farms.

**Conclusions:**

Therefore, mosquitoes appear unlikely to transmit ASFV, and pose little threat to large-scale pig farms. Thus, farms should continue to follow normal mosquito control procedures when formulating strategies for the prevention and control of ASF.

## Introduction

African swine fever (ASF) causes great economic losses to the pig industry due to its rapid spread, infectiousness, and high mortality. ASF was first reported in 1921 in domestic pigs in Kenya, and it had spread to Europe in 1957, followed by Ukraine, Lithuania, Latvia, Russia, and various other countries between 2012 and 2017. Since August 2018, ASF has been prevalent in China, causing serious economic losses to the swine industry [1]. At present, no safe and effective vaccine for ASF has been successfully developed, and infection sources and transmission routes of ASF virus (ASFV) are many and complex due to its resistance to the external environment. It remains infectious for a long time in a variety of porcine products, arthropods, and contaminated environments, greatly increasing the difficulty of its prevention and control [2]. Therefore, timely detection, containment of sources of infection, and blockage of transmission are important for preventing the spread of ASF. Soft ticks are the main vectors and reservoir hosts of ASFV, and ASF is mainly circularly transmitted in three ways; pig-pig, pig-soft tick, and pig-soft tick-wild boar contacts [3]. ASFV replicates in soft ticks of the ornithodoros genus that become a reservoir of the virus, causing infection by biting pigs or by feeding. Soft ticks facilitate viral replication, remain infected up to the fourth week after feeding, and can still infect pigs after 469 days of infection. However, although soft ticks are a potential source of ASFV infection, there is currently no evidence of a direct relationship between soft ticks and the transmission of ASFV in Europe [4, 5]. Additionally, although soft ticks are distributed throughout China, they have great geographical environmental limitations compared with other sources of vector-borne transmissions such as mosquitoes.

As the climate in southern China gets hotter and the season for active mosquitoes arrives, mosquitoes may carry and transmit ASFV in pig farms, but this has not been proven. Mosquitoes are known to transmit as many as 80 diseases through blood feeding, including Japanese encephalitis virus, West Nile virus, dengue virus, Zika virus, or yellow fever virus [6]. The occurrence of ASF is highly seasonal in Europe, and mainly endemic from July to September, which may be related to the frequent activity of blood-feeding insects (e.g., flies, mosquitoes) in summer [7]. One research group captured 15 different species of insects (including house flies and mosquitoes) and identified nucleic acids from house flies and mosquitoes in an epidemiological survey following an ASF outbreak [8]. These studies indicated the possibility of mosquito transmission of ASFV, but there are no relevant reports in China. Therefore, in this study, we measured CT values for ASFV by probe-based qPCR in mosquitoes captured from five large-scale pig farms with ASF, or suspected of having ASF, in Jiangsu Province, China. We investigated the possible mosquito carriage of ASFV, and the potential threat to large-scale pig farms. The results could inform the development of strategies for the prevention and control of ASF in pig farms.

## Materials and methods

### Mosquito sample collection

A total of 463 mosquitoes were captured from pigpens in five large-scale pig farms with ASF, in Jiangsu Province (Table 1). Fifty to one hundred mosquitoes were captured using mosquitoes trap cage from each pig farm, the mosquitoes were identified by stereo microscope. After a week of collection, all mosquitoes were cleaned using 75 % ethanol for 5 min, rinsed twice in sterile phosphate-buffered saline (PBS), and immediately placed in liquid nitrogen for storage.

**Table 1:** The number of captured mosquitoes from five pig farms

city of pig farms	current status	number of mosquitoes
Changzhou	during the outbreak	89 (3 Anopheles)
Nantong	during the outbreak	94
Yangzhou	2 weeks after the outbreak	103
Liyang	1 month after the outbreak	89 (5 Aedes)
Yixing	2 months after the outbreak	88

### Extraction of viral DNA from mosquitoes and identification of ASFV

DNA from half of individual mosquito was extracted using an Ascend nucleic acid extraction kit (Luoyang Ascend Biotechnology Co., LTD, China). The concentration and purity of DNA were determined using the Nanodrop ONE spectrophotometer. According to Protocol of Quarantine for African Swine Fever (SN/T 1559–2010) enacted by the General Administration of Quality Supervision, Inspection and Quarantine of the People’s Republic of China, probe-based qPCR was used to detect virus from individual mosquitoes, forward primers (50 pmol/µL) 5’-CTGCTCATGGTATCAATCTTATCGA-3’ and reverse primer (50 pmol/µL) 5’-GATACCACAAGATC (A/G) GCCGT-3’, probe 5’-CCACGGGAGGAATACCAACCCAGTG-3’ (5 pmol/µL). In this analytical method, carboxyl fluorescein (FAM) and tetramethyl-6-carboxyrhodamine (TAMRA) were respectively selected as fluorophore of aptamer probes. after which the remaining mosquitoes from same farm were mixed prior to DNA extraction. Samples were considered positive with a CT value ≤ 35, potentially positive with a CT value between 35 and 40, and negative with no CT value. For potentially positive samples the amplification curve was inspected, and if logarithmic amplification was observed samples were presumed positive, but they were presumed negative if amplification was not logarithmic. DNA was re-extracted and probe-based qPCR assays were performed on these suspicious samples, and if the repeated amplification curves showed logarithmic amplification, samples were considered positive sample, but they were considered negative if amplification was not logarithmic. The positive control was standard ASFV strain (500ng/µL Pig/HLJ/18 DNA was provided by Animal Disease Testing and Technical Service Center of Yangzhou University (ADTTSC)), and the negative control was sterilized distilled water without DNA.

## Results

### Identification of mosquito species

**Fig. 1:**
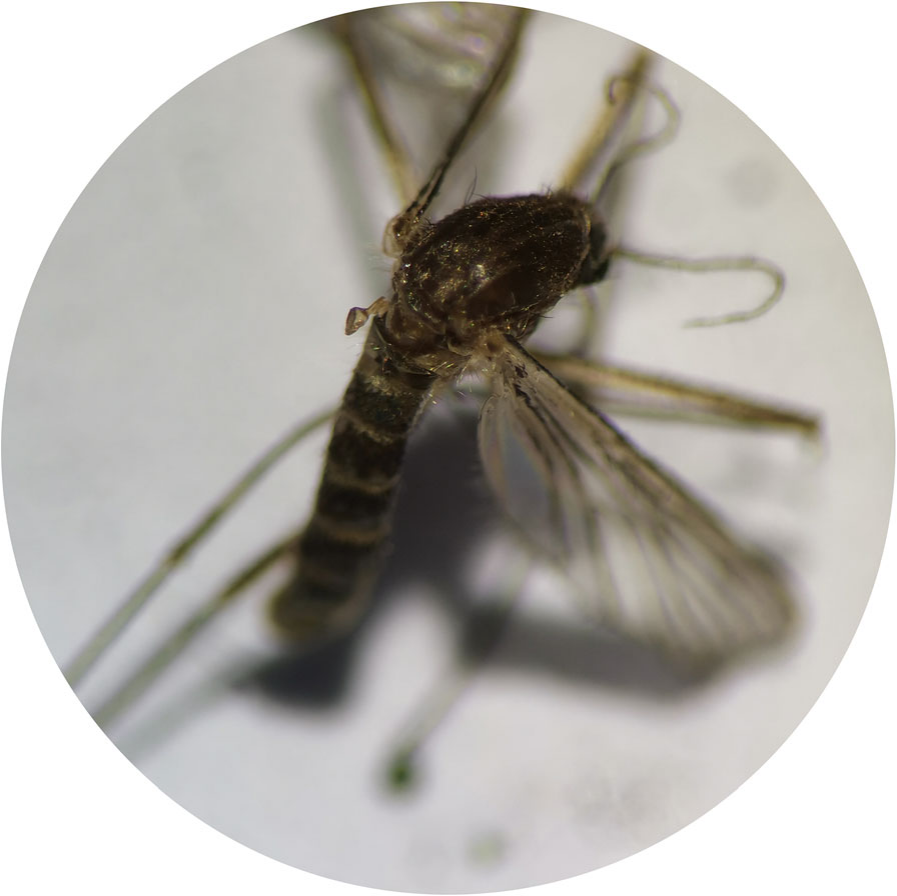
Microscopic enlargement of mosquitoes in pig farms affected by ASF

In this study, mosquito species were determined by stereomicroscopy, and the results revealed most of them to be *Culex pipiens pallens* (455 *Culex pipiens pallens*) (Fig. 1), there were only a few Anopheles and Aedes mosquitoes (3 Anopheles, 5 Aedes) in accordance with the distribution of common mosquitoes in Jiangsu Province [9]. In the following study, we discarded Anopheles and Aedes mosquitoes due to the small numbers.

### *ASFV detection by* probe‐based qPCR

**Fig. 2:**
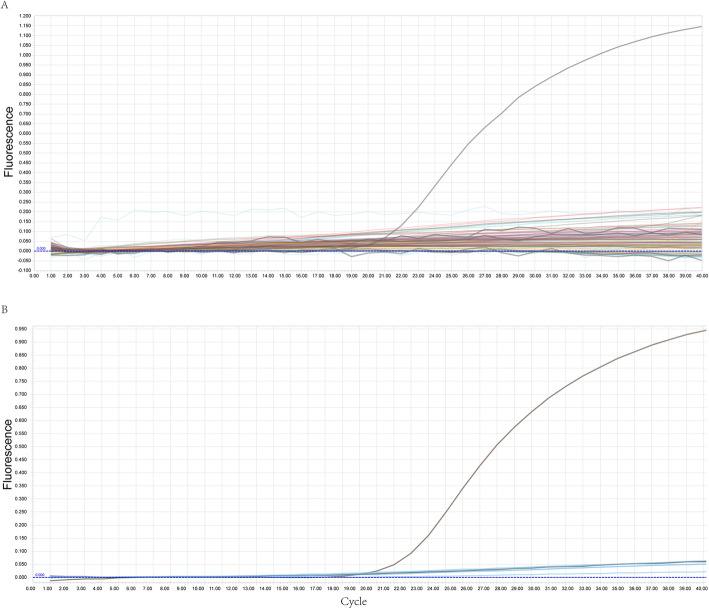
Probe-based qPCR analysis of ASFV in mosquitoes. **a** probe-based qPCR detection for each mosquito, **b** probe qPCR detection for mosquitoes from each pig farm

probe-based qPCR was performed on 463 mosquitoes from five pig farms, CT value of positive control was 21.54, while the mosquitoes in all five farms gave negative results (no CT values; Fig. 2 a). This may be because a single mosquito carrying a small amount of virus could not be detected. Thus, the remaining mosquitoes from the same pig farm were mixed and DNA was extracted, but the results remained negative (no CT values; Fig. 2b).

## Discussion

ASFV, a complex, cytoplasmic double-stranded DNA virus 175−215 nm in diameter, is the only DNA virus that uses arthropods as biological vectors [10]. At present, our understanding of ASFV is not comprehensive, and many aspects including virulence, pathogenesis and transmission mechanism remain poorly understood. The development of safe and effective ASVF vaccines may take a long time, hence understanding potential vectors is essential for preventing epidemic outbreaks and detecting potential epidemics. Thus, it is necessary to investigate the possibility of mosquitoes as ASFV carriers and biological or mechanical vectors.

In this study, five pig farms in Jiangsu Province with ASF, or suspected of having ASF, were selected, 463 mosquitoes were captured, and they were confirmed to be *Culex pipiens pallens* by stereomicroscopic observation. A previous study counted the constituent ratio of mosquitoes in Jiangsu Province from April to November and found that *Culex pipiens pallens* was the dominant species, with indoor captures accounting for 75.07−92.86 % of all mosquitoes [9], consistent with the results of the present study.

A number of criteria are usually met when judging whether or not mosquitoes are virus vectors; (1) viruses can be isolated from mosquitoes; (2) under experimental conditions, mosquitoes can be infected after feeding on blood of the viral host; (3) mosquitoes can transmit the virus by biting vertebrate host during the experiment; (4) mosquitoes feed on blood from natural hosts and this can be proven [11]. If mosquitoes meet the above four criteria, mosquitoes can be considered a vector of the virus. The mosquitoes detected in the present study yielded negative results for ASFV, and did not satisfy criteria 1.

There have been very few studies on the potential role of mosquitoes in ASFV transmission, but one failed to validate the vector competence of mosquitoes and horse flies for the virus [12]. A recent study detected low viral doses in mosquitoes collected from an infected farm in Estonia, but virus isolation was unsuccessful, and the researchers believe that the viral DNA found in mosquitoes is likely to be due to general contamination of the environment by ASFV, but whether mosquitoes have transmission capacity requires further study [8]. The mosquitoes detected in the present study yielded negative results for ASFV, we speculate that the mosquitoes do not have the ability to transmit ASFV, that the blood detected in Herm’s study may have come from the host, and that the blood fed by the mosquitoes in this study may have been digested. Generally, viruses cannot survive alone and die after a period of time after leaving the host, but the lifespan of different viruses in mechanical vectors is inconsistent. African swine fever virus was transmitted to susceptible pigs by flies infected one hour and 24 h previously and the virus survived in these flies for at least two days without apparent loss of titre [13]. This may be the reason for the negative results of this study. Along with the present work, none of the above reports could confirm the role of mosquitoes as vectors in transmitting ASFV. Epidemiological surveys in China have shown that among the 155 ASFV outbreaks in which the epidemic source has been identified, 100 outbreaks have been caused by the allocation and transportation of pigs and their products, and the transmission of viruses by related personnel and vehicles, accounting for ~ 64.5 % [14].

The transmission of ASF mainly depends on its strong resistance, since the virus can survive in the external environment and various biological media for a long time. Vehicles, personnel, tools and swill are the main sources of virus transmission. Therefore, we speculate that mosquitoes are not heavily involved in ASFV transmission, but the possibility of mechanical transmission cannot be ruled out. Large-scale pig farms still need to follow normal mosquito control procedures when formulating strategies for the prevention and control of ASF, but new virus-specific measures are not needed. Rather, attention should be payed to disinfection and epidemic prevention management related to personnel and vehicles entering and leaving farms.

## Data Availability

The datasets used and analyzed during the current study available from the corresponding author on reasonable request.

## Abbreviations

**ASFV**: African swine fever virus.
**CT**: cycle threshold.
**PBS**: phosphate-buffered saline.
